# Effect of BBT-877, a novel inhibitor of ATX, on a mouse model of type 1 diabetic nephropathy

**DOI:** 10.18632/aging.204249

**Published:** 2022-08-26

**Authors:** Jong Han Lee, Phyu Phyu Khin, GwangHee Lee, Oh Kyung Lim, Hee-Sook Jun

**Affiliations:** 1 Department of Marine Bio and Medical Science, Hanseo University, Seosan, Korea; 2 College of Pharmacy and Gachon Institute of Pharmaceutical Science, Gachon University, Incheon, Korea; 3 Bridge Biotherapeutics Incorporation, Seongnam, Korea; 4 Boostimmune Therapeutics Incorporation, Seongnam, Korea; 5 Department of Rehabilitation Medicine, Gachon University, Incheon, Korea; 6 Lee Gil Ya Cancer and Diabetes Institute, Gachon University, Incheon, Korea

**Keywords:** streptozotocin-induced diabetic mice, BBT-877, BBT-877, autotaxin, diabetic nephropathy

## Abstract

Diabetic nephropathy (DN) is one of the common microvascular complications of diabetes. Autotaxin (ATX) is an enzyme with lysophospholipase D activity, producing lysophosphatidic acid (LPA). LPA signaling has been implicated in renal fibrosis, thereby inducing renal dysfunction. BBT-877 is an orally administered small molecule inhibitor of ATX. However, its effect on DN has not been studied so far. In this study, we investigated the effect of BBT-877, a novel inhibitor of ATX, on the pathogenesis of DN in a mouse model of streptozotocin (STZ)-induced diabetes.

BBT-877 treatment significantly reduced albuminuria, albumin-to-creatinine ratio (ACR), neutrophil gelatinase-associated lipocalin (NGAL), and glomerular volume compared to the STZ-vehicle group. Interestingly, BBT-877 treatment attenuated hyperglycemia and dyslipidemia in STZ-induced diabetes mice. In the liver, the expression levels of β-oxidation-related genes such as PPAR α and CPT1 were significantly decreased in STZ-induced diabetic mice. However, this effect was reversed by BBT-877 treatment. BBT-877 treatment also suppressed mRNA levels of pro-inflammatory cytokines IL-6, MCP-1, and TNF-α and protein levels of fibrotic factors (TGF-β, fibronectin, CTGF, and collagen type Ι alpha Ι (COL1A1)) in the kidneys of STZ-induced diabetic mice.

In conclusion, our results indicate that BBT-877 is effective in preventing the pathogenesis of DN by reducing systemic blood glucose levels and inhibiting inflammation and fibrosis in the renal tissue of diabetes mice. These novel findings suggest that inhibition of ATX may be a potential therapeutic target for DN.

## INTRODUCTION

A progressive kidney condition called diabetic nephropathy (DN) is one of the common microvascular complications of diabetes and frequently leads to end-stage renal disease (ESRD) in individuals with diabetes. Proteinuria, diabetic glomerular lesions, and a reduction in glomerular filtration rate are well-known pathological features of DN [1, 2]. Hyperglycemia, hypertension, dyslipidemia, and genetic predisposition are major contributors to the development of DN [3, 4]. Hyperglycemia causes renal damage through a variety of mechanisms, including the production of advanced glycation end products and the activation of their receptors, reactive oxygen species generation, and inflammation [5, 6]. In particular, renal inflammation and fibrosis are important cellular and molecular mechanisms in the pathogenesis of DN [7–9]. Therefore, glycemic control is a well-known treatment for DN [1, 10].

Autotaxin (ATX) is an enzyme with lysophospholipase D activity, producing lysophosphatidic acid (LPA) from lysophospholipids. ATX was originally isolated from a conditioned medium of A2058 human melanoma cells [11] and was later identified as the sole source of extracellular LPA [12]. LPA is a small phospholipid derivative that mediates various cellular processes, such as proliferation, survival, and migration, via its G protein-coupled receptors, LPAR1-6s [13]. In addition, LPA induces cell damage through complex overlapping pathways mediated by the generation of reactive oxygen species, inflammatory cytokines, and fibrosis [14, 15]. In particular, the LPA-LPAR axis plays an important role in the pathogenesis of kidney disease, lung fibrosis, and cancer [16]. Indeed, LPA is increased in the serum and kidney cortex of db/db mice, which are used to model type 2 diabetes and obesity [17, 18]. Similarly, ATX is increased in the fat and kidney tissues of db/db mice [19, 20]. A recent study showed that LPA is significantly increased in the urine of patients with type 2 diabetes exclusively associated with albuminuria [21]. We have also reported that the LPA-LPAR axis can induce pathological alterations in the structure and function of kidney cells by upregulation of renal inflammation and fibrosis [15]. In contrast, LPAR antagonism improved renal function in DN through various mechanisms, such as suppression of oxidative stress and inflammation [15, 22, 23]. ATX inhibition is another potential approach to suppress the LPA-LPAR signaling cascade that may have a beneficial effect on the pathogenesis of DN, similar to the effect of LPAR antagonism.

BBT-877 and PF-8380 are orally available small molecule inhibitors of ATX. Recently, Lee et al. showed that BBT-877 treatment effectively decreased LPA production in *in vitro* and *ex vivo* systems [24]. In addition, several ATX inhibitors, including BBT-877, suppressed fibrosis in bleomycin-induced pulmonary fibrotic mice [25] and in liver fibrotic mice [26]. In particular, BBT-877 was less cytotoxic to various cell types, such as hepatocellular carcinoma cells and normal lung fibroblasts [24]. These exciting observations indicate that BBT-877 may have a potential therapeutic effect on DN by suppressing the ATX-LPA-LPAR signaling-mediated renal inflammation and fibrosis. However, the effect of BBT-877 on DN has not been investigated so far.

In this study, we investigated the effect of BBT-877, a novel inhibitor of ATX, on the pathogenesis of DN in a mouse model of streptozotocin (STZ)-induced diabetes. Our results demonstrated that BBT-877 administration restored kidney dysfunction in diabetic mice by lowering systemic blood glucose and lipid levels as well as reducing inflammation and fibrosis in the kidney.

## RESULTS

### BBT-877 treatment attenuates hyperglycemia and dyslipidemia in STZ-induced diabetic mice

**Figure 1:**
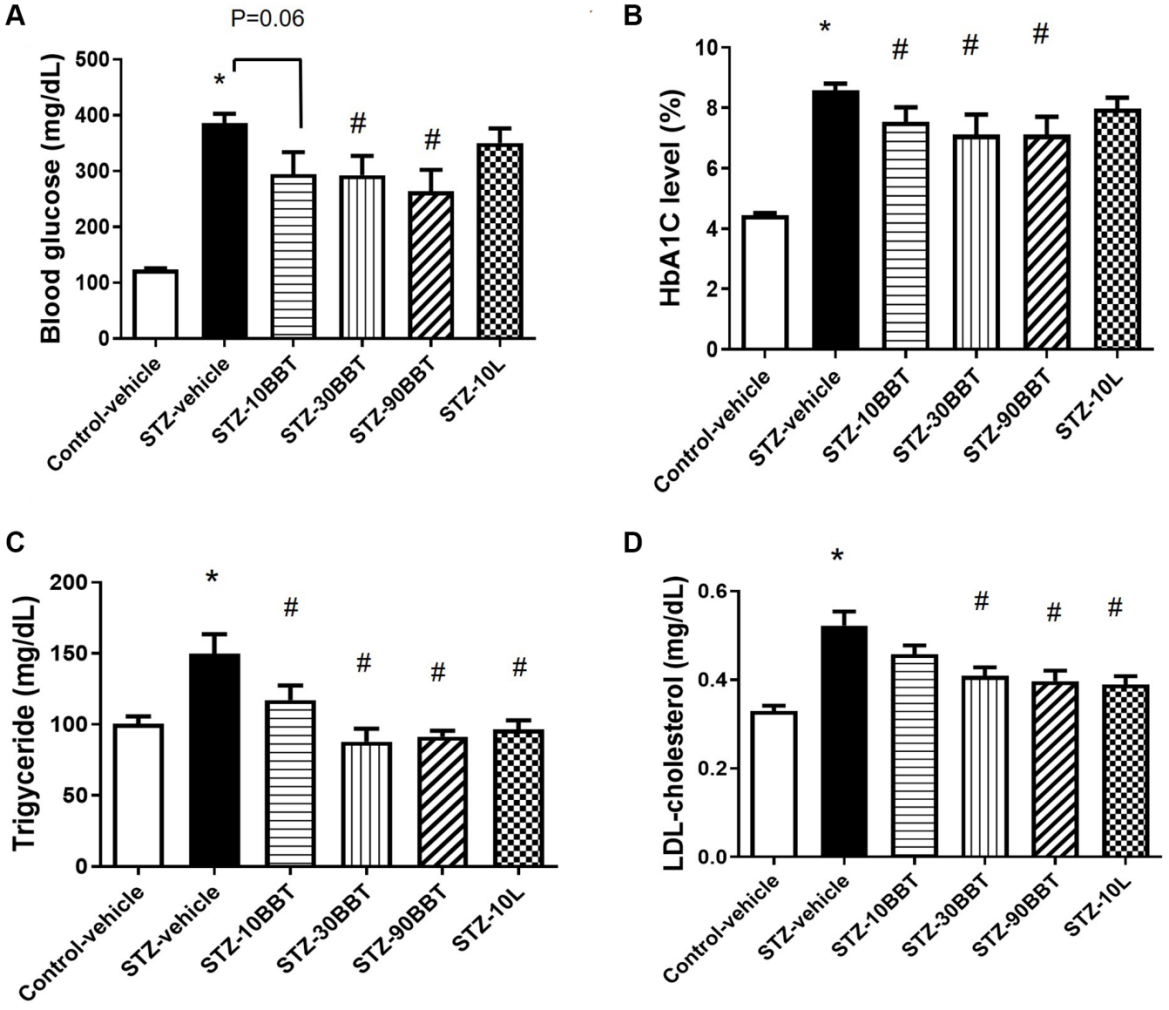
**BBT-877 attenuates hyperglycemia and dyslipidemia in STZ-induced diabetic mice.** After mice were administered BBT-877 for 8 weeks, measurements were taken for (**A**) blood glucose, (**B**) HbA1c, (**C**) Triglyceride, and (**D**) LDL-cholesterol. Abbreviations: STZ: streptozotocin; HbA1c: glycosylated hemoglobin. STZ-10BBT: BBT-877 10 mg/kg, STZ-30BBT: BBT-877 30 mg/kg, STZ-90BBT: BBT-877 90 mg/kg, STZ-10L: losartan 10 mg/kg. ^*^p < 0.05, control-vehicle vs. STZ-vehicle, ^#^*p* < 0.05, STZ-vehicle vs. STZ-10, 30, 90BBT or STZ-10L, *n* = 8−10.

Body weight significantly decreased in the STZ-vehicle group compared to the control-vehicle group, but this effect was ameliorated by BBT-877 and losartan administration (Table 1). Blood glucose was also remarkably increased in the STZ-vehicle group compared to the control-vehicle group, but this effect was significantly decreased by BBT-877 treatment in a dose-dependent manner (Figure 1A). Although the losartan group had slightly lower blood glucose levels than the STZ-vehicle mice, the effect was not significant (Figure 1A). The levels of HbA1c displayed a similar trend to that of blood glucose levels, with a dose-dependent response in the BBT-877-treated groups (Figure 1B). In addition, the losartan group had slightly lower HbA1c levels than the STZ-vehicle mice, but the effect was not significant (Figure 1B). Triglyceride and cholesterol levels were significantly increased in the STZ-treated mice (Figure 1C, 1D), and this effect was ameliorated in a dose-dependent manner by BBT-877 administration, and similarly by losartan treatment (Figure 1C, 1D). Furthermore, the weights of tissues, such as the kidney and spleen, which were measured at the end of the experiment, were comparable among the treatment groups (control, STZ-vehicle, and STZ-BBT-877), except for the liver (Table 1). Although the weight of the liver was increased in the STZ-vehicle group, it decreased in a dose-dependent manner following treatment with BBT-877 (Table 1). In STZ-induced diabetic mice, β-oxidation-related genes of peroxisome proliferator-activated receptor α (*PPAR* α) and carnitine palmitoyltransferase-1 (*CPT1*) were significantly reduced in the liver. However, this effect was reversed by BBT-877 treatment (Supplementary Figure 1). In contrast, the expression levels of the lipogenesis-related genes—fatty acid synthase (FAS) and stearoyl-CoA desaturase-1 (SCD-1)—did not show any statistically significant differences across all groups. Interestingly, the expression of the fatty acid binding protein 4 (FABP4)—a potential biomarker for fat accumulation—significantly increased and reduced by BBT-877 in a dose-dependent manner (Supplementary Figure 1).

**Table 1:** Effect of BBT-877 treatment on physiological parameters of STZ-induced diabetic mice.

**Characteristic**	**CV**	**SV**	**S108**	**S308**	**S908**	**S10L**
Body weight (g)	26.9 ± 0.7	23.5 ± 0.4^†^	25.0 ± 0.4	24.4 ± 0.4	24.5± 0.4	25.0 ± 0.3
Kidney weight (mg)	297.3 ± 8.5	287.7 ± 5.8	272.8 ± 10.3	286.0 ± 11.1	283.4 ± 6.3	292.8 ±8.7
Liver weight (mg)	1105± 40	1385 ± 59^†^	1255 ± 48	1157 ± 57^‡^	1219 ± 46^‡^	1227 ± 48^‡^
Spleen weight (mg)	54.2 ± 3.16	55.4 ± 3.8	49.3 ± 1.9	47.4 ± 2.1	54.3 ± 2.4	48.4 ± 1.0
Food intake (gig per day)	0.19 ± 0.02	0.30 ± 0.03^†^	0.29 ± 0 .03	0.28 ± 0.03	0.26 ± 0.03	0.31 ± 0.03
Water intake (m/lg per day)	0.014 ± 0.0	0.064 ± 0.0^†^	0.045 ± 0.1^*p* = 0.08^	0.044 ± 0.0	0.039 ± 0.0^‡^	0.044 ± 0.0^‡^
Urine volume (ml/g per day)	0.046 ± 0.0	0.501 ± 0.1^†^	0.282 ± 0.1^*p* = 0.08^	0.270 ± 0.1^*p* = 0.07^	0.252 ± 0.1^‡^	0.329 ± 0.1
Albumin/creatinine ratio (μg/mg)	6.87 ± 0.5	31,94 ± 2.9^†^	21.27 ± 3.4^‡^	21.19 ± 4.3^*p* = 0.06^	19.36± 3.5^‡^	25.66 ± 3.6

Albumin/creatinine ratio was measured at 4 weeks; other parameters were measured at 8 weeks. Data are displayed as mean ± SEM. Abbreviations: STZ: streptozotocin; HbA1c: glycosylated hemoglobin; CV: control-vehicle; SV: STZ-vehicle; S10B: STZ-10 mg BBT-877; S30B: STZ-30 mg BBT-877; S90B: STZ-90 mg BBT-877; S10L: STZ-10 mg Losartan. ^†^*p* < 0.01 vs. CV; ^‡^*p* < 0.05 vs. SV, noted *p* values are vs. SV (*n* = 8−10).

### BBT-877 treatment improves kidney function in STZ-induced diabetic mice

Food intake was comparable among STZ groups but showed a tendency to decrease in a dose-dependent manner in the STZ-BBT-877 groups, although the difference was not statistically significant (Table 1). Urination and increased water intake were reduced by BBT-877 in a dose-dependent manner in diabetic mice at 4 weeks, compared to the STZ-vehicle group (Table 1). In particular, the reduction in water intake and urination was significant in mice administered with 90 mg/kg BBT-877 (Table 1). Consistently, losartan exerted a similar effect on both parameters, as shown in Table 1. compared to the control-vehicle group, the microalbumin and creatinine ratio (ACR) increased in the diabetic mice at 4 weeks. In contrast, treatment with BBT-877 decreased the ACR ratio in all diabetic groups. The reduction levels were similar and reached statistical significance at both 10 and 90 mg/kg BBT-877 (Table 1). Losartan administration lowered the ACR compared to the STZ-vehicle group but the change was not significantly different (Table 1). Consistently, the concentration of urinary neutrophil gelatinase-associated lipocalin (NGAL) was highly increased in diabetic mice. However, treatment with BBT-877 significantly suppressed the release of NGAL in a dose-dependent manner, indicating that BBT-887 can reverse the diabetes-induced tubular damage of the kidney. In addition, the protein and mRNA expression levels of NGAL were increased in the STZ-vehicle group but suppressed by BBT-877 and losartan, as positive control treatment (Supplementary Figure 2).

### BBT-877 treatment reduces glomerular injury in the kidneys of STZ-induced diabetic mice

**Figure 2:**
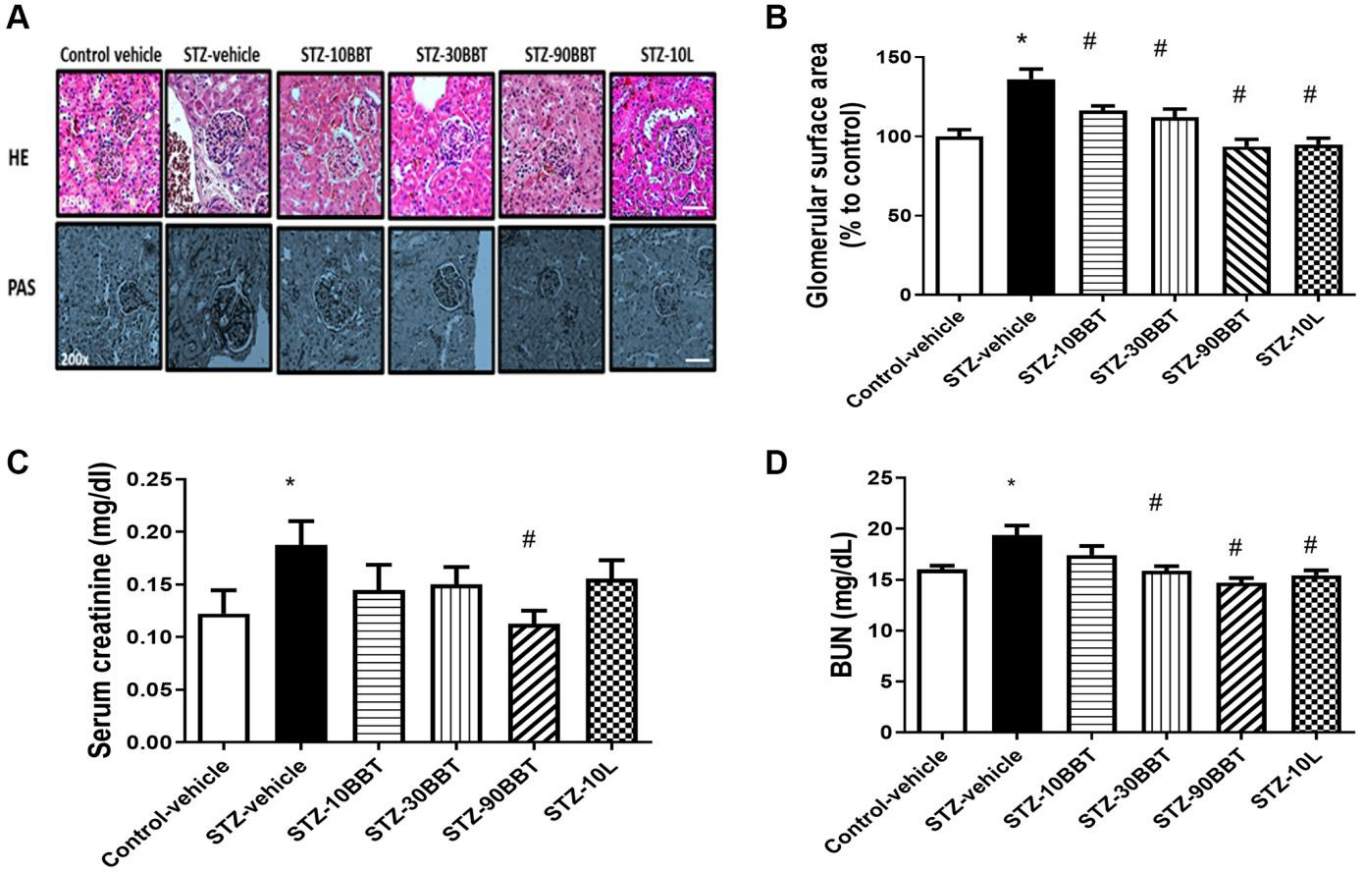
**BBT-877 reduces glomerular injury in the kidneys of STZ induced diabetic mice.** After treatment with BBT-877 for 8 weeks, mice were sacrificed and their kidneys were collected, fixed in 10% formalin buffer, and embedded in paraffin. The kidney sections were stained with hematoxylin and eosin (HE) and Periodic acid-Schiff (PAS). Glomerular surface area was quantified using 30 glomeruli per mouse by ImageJ Software. (**A**) Representative image of HE and PAS staining, scale bars = 20 μm (**B**) Glomerular surface area, (**C**) Serum creatinine, (**D**) Blood urea nitrogen. Abbreviation: STZ: streptozotocin. STZ-10BBT: BBT-877 10 mg/kg, STZ-30BBT: BBT-877 30 mg/kg, STZ-90BBT: BBT-877 90 mg/kg, STZ-10L: losartan 10 mg/kg. ^*^p < 0.05, control-vehicle vs. STZ-vehicle, ^#^*p* < 0.05, STZ-vehicle vs. STZ-10, 30, 90BBT or STZ-10L, *n* = 8−10.

The glomerular surface area significantly increased in diabetic mice. However, this increase was remarkably decreased by treatment with BBT-877 in a dose-dependent manner (Figure 2A, 2B). Furthermore, the glomerular surface area was comparable between the high-BBT-877 and losartan treatment groups (Figure 2A, 2B). Since creatinine and BUN are known biomarkers of renal function, we measured their serum levels after 8 weeks of BBT-877 administration. Serum creatinine levels were increased in STZ-vehicle mice but decreased by BBT-877. Furthermore, a statistically significant difference was observed following the administration of 90 mg/kg BBT-877 (Figure 2C). Similarly, 10 mg/kg losartan attenuated this increase, but did not differ significantly from the STZ-vehicle group (Figure 2C). BUN levels were also increased in the STZ-vehicle group compared to the control-vehicle mice, and this was also significantly decreased by BBT-877 and losartan (Figure 2D).

### BBT-877 treatment inhibits the expression of pro-inflammatory cytokines and fibrotic factors in the kidneys of STZ-induced diabetic mice

**Figure 3:**
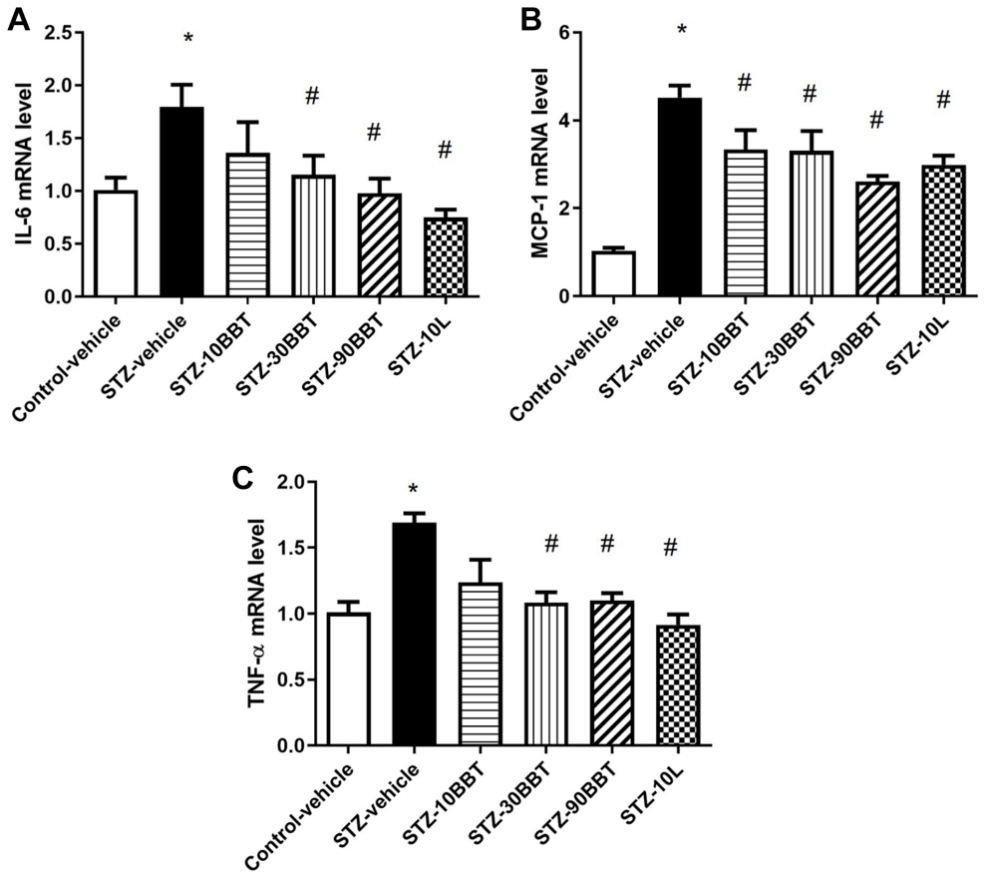
**BBT-877 reduces the mRNA levels of inflammatory cytokines in STZ-induced diabetic mice.** After treatment with BBT-877 for 8 weeks, mice were sacrificed and their kidneys were collected. RNA was extracted and subjected to qRT-PCR for (**A**) IL-6, (**B**) MCP-1, and (**C**) TNF-α. Abbreviations: STZ: streptozotocin; qRT-PCR: quantitative reverse transcriptase-polymerase chain reaction. STZ-10BBT: BBT-877 10 mg/kg, STZ-30BBT: BBT-877 30 mg/kg, STZ-90BBT: BBT-877 90 mg/kg, STZ-10L: losartan 10 mg/kg. ^*^p < 0.05, control-vehicle vs. STZ-vehicle, ^#^*p* < 0.05, STZ-vehicle vs. STZ-10, 30, 90BBT or STZ-10L, *n* = 8−10.

**Figure 4:**
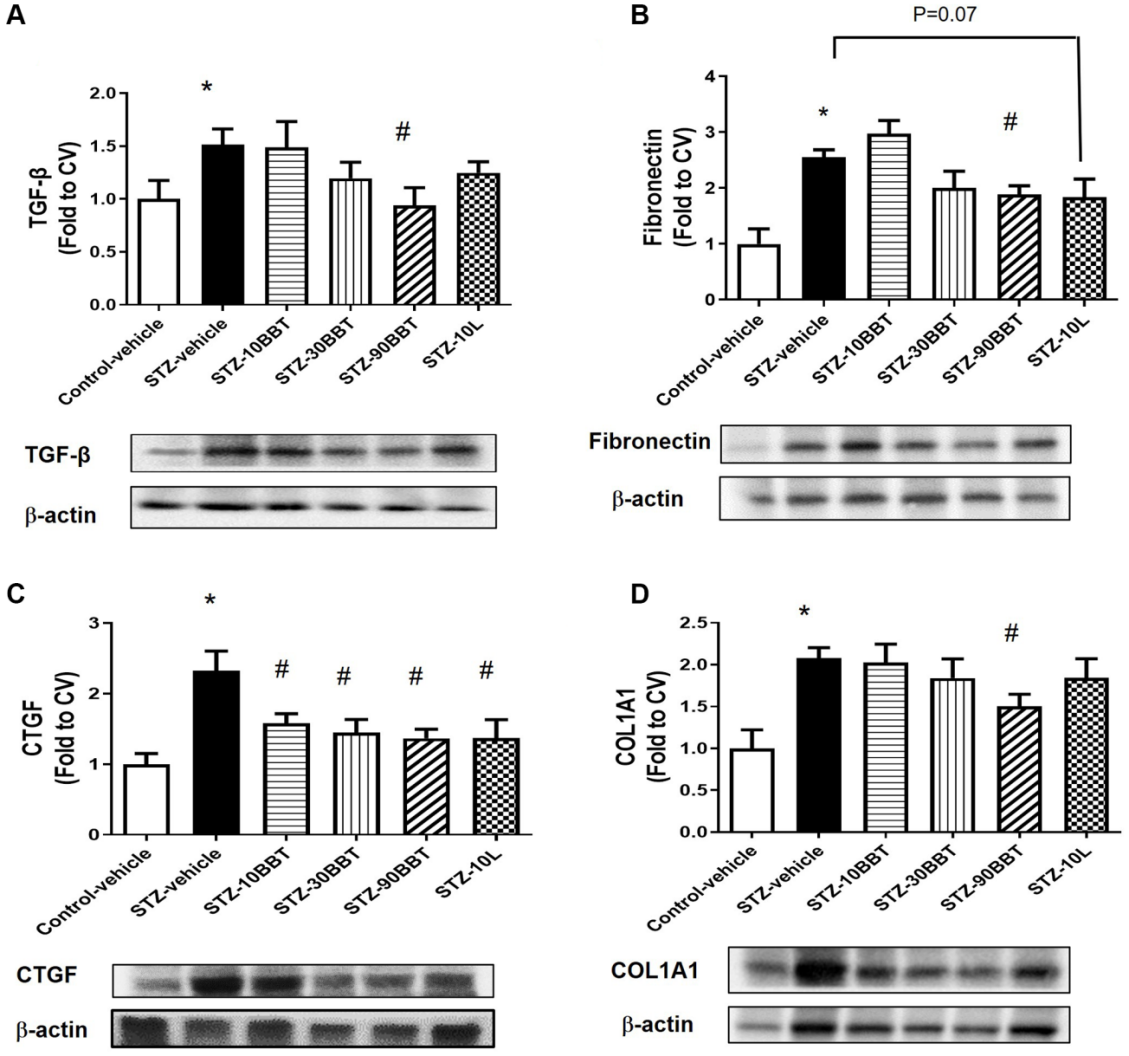
**BBT-877 reduces the protein levels of fibrotic factors in STZ-induced diabetic mice.** After treatment with BBT-877 for 8 weeks, mice were sacrificed and their kidneys were collected. Protein was extracted and subjected to western blotting for (**A**) TGF-β, (**B**) Fibronectin, (**C**) CTGF, and (**D**) COL1A1. Abbreviations: STZ: streptozotocin; qRT-PCR: quantitative reverse transcriptase-polymerase chain reaction. STZ-10BBT: BBT-877 10 mg/kg, STZ-30BBT: BBT-877 30 mg/kg, STZ-90BBT: BBT-877 90 mg/kg, STZ-10L: losartan 10 mg/kg. ^*^p < 0.05, control-vehicle vs. STZ-vehicle, ^#^*p* < 0.05, STZ-vehicle vs. STZ-10, 30, 90BBT or STZ-10L, *n* = 8−10.

To determine whether BBT-877 treatment modulates the expression of pro-inflammatory cytokines and fibrotic factors in the kidneys of STZ-induced diabetic mice, we measured the mRNA expression of inflammatory cytokines and the protein expression of fibrotic factors. The expression levels of interleukin 6 (IL-6), tumor necrosis factor-α (TNF-α), and monocyte chemoattractant protein-1 (MCP-1) were significantly increased in STZ-induced diabetic mice; however, this increase was suppressed in a dose-dependent manner by BBT-877 administration (Figure 3). Losartan treatment also suppressed inflammatory cytokine expression to a level similar to the highest dose of BBT-877 (Figure 3). Consistent with these findings, TGF-β expression level was increased in STZ-induced diabetic mice; however, this increase was reduced by BBT-877, again in a dose-dependent manner, with a statistically significant difference observed with 90 mg/kg BBT-877 (Figure 4A). Similarly, the expression level of fibronectin was upregulated in the STZ-vehicle group compared to the control-vehicle group. However, both BBT-877 (90 mg/kg) and losartan (10 mg/kg) remarkably suppressed its expression (Figure 4B). The expression level of CTGF was significantly reduced in all treatment groups, including the losartan group, compared to the STZ-vehicle group (Figure 4C). Moreover, COL1A1 protein expression levels were significantly decreased by treatment with 90 mg BBT-877 (Figure 4D).

## DISCUSSION

We and others have shown that LPAR antagonism improves renal dysfunction in both type 1 and type 2 diabetic rodent models [15, 22, 23]. Here, we demonstrated that BBT-877, a novel inhibitor of ATX, attenuates DN in type 1 diabetic mice by lowering systemic blood glucose levels as well as suppressing the expression of pro-inflammatory cytokines and fibrotic factors in the kidney.

ATX is an enzyme responsible for LPA production. The ATX-LPA-LPAR axis is involved in the pathogenesis and progression of DN, including glomerulosclerosis, tubulointerstitial fibrosis (TIF), and changes in renal vasculature [27]. Serum ATX and LPA levels are associated with increased proteinuria and kidney failure in type 2 diabetes patients [28]. In addition, LPAR1-4 are expressed in kidney tissue under normal conditions, and LPAR 1 and 3 are further elevated in diabetic conditions [22]. Our previous studies and those of others demonstrated that inhibition of the LPA-LPAR axis by LPA receptor antagonists (such as ki16425, AM095, and BMS002) attenuates kidney dysfunction through various cellular and molecular mechanisms, including anti-oxidant, anti-inflammatory, and anti-fibrotic effects [15, 22, 23].

BBT-877 is an orally available small molecule inhibitor of ATX that mimics LPA receptor antagonism, leading to the suppression of fibrosis in lung tissue [24, 29]. Our current data reveal that BBT-877 administration similarly attenuates kidney hyperfiltration and kidney dysfunction in diabetic states, as revealed by the reduced water intake, urination, and ACR compared to the STZ-vehicle group, respectively. These data are consistent with a study showing that another ATX inhibitor, PF-8380, ameliorated progressive proteinuria and TIF in chronic allograft injury in rats [30].

Consistently, the biomarkers of kidney dysfunction, including serum creatinine, BUN, and urinary NGAL, improved in a dose-dependent manner, while glomerular volume reduced in the BBT-877-treated STZ-induced diabetic mice. Additionally, BBT-877 reduced blood glucose and HbA1c levels without changing the body weight of mice. These findings could explain how the administration of BBT-877 recovers kidney dysfunction. Similarly, several studies have shown that glycemic control significantly attenuates microalbuminuria and proteinuria, as well as reduces kidney dysfunction in diabetes patients [31–33]. In alignment with the previous results from our group and others, losartan did not attenuate the blood glucose and HbA1c levels, despite the reduction trend identified following its administration [15, 22, 34].

In ATX null and adipocyte-specific ATX knockout mice, they showed smaller body weight gains and less insulin resistance under high fat diet feeding by suppressing adipose tissue expansion [35]. Similarly, lowering LPA level by BBT-877 administration may indirectly affect to key peripheral tissues associated with systemic insulin sensitivity, including the liver, fat tissue, and pancreas. Indeed, BBT-877 treatment group mice had significantly lighter liver than SV group mice. The expression levels of several genes in the liver (such as *PPAR α, CPT1* involved in β-oxidation, and *FABP4* involved in triglyceride synthesis and its accumulation) in the SV group indicated fat accumulation. This possibly impairs liver function associated with glycemic control.

In contrast, the levels of triglyceride and LDL-cholesterol were significantly reduced in the BBT-877 treatment groups compared to that in the SV group. Reduced TG and LDL-cholesterol in serum by BBT-877 treatment may also enhance systemic insulin sensitivity by attenuating lipotoxicity in peripheral tissues and/or by suppressing systemic and local inflammation levels. Collectively, these beneficial effects of BBT-877 on insulin sensitivity may attenuate DN. However, the current study does not cover the detailed molecular mechanism by which BBT-877 regulates blood glucose and lipid levels. Therefore, further studies are needed to elucidate the precise molecular mechanisms involved in key peripheral tissues.

Inflammatory cytokines and fibrotic factors play important roles in the progression of DN [22, 36, 37]. Although fibrosis allows normal wound healing and injury repair in the maintenance of various normal tissues [38], acute or chronic injurious stimuli can cause a dysregulation of normal healing processes. This can lead to excess deposition of the extracellular matrix and fibrosis, and a complex multistage inflammatory process, which ultimately results in cell death and tissue injury [39, 40]. Several studies have shown that activation of the LPA-LPAR axis increases the expression of renal inflammatory cytokines [16, 22, 41]. Similarly, diabetic mice had significantly higher mRNA expression levels of IL-6, TNF-α, and MCP-1 than control mice. However, these levels were significantly suppressed by BBT-877 treatment.

TGF-β is the central cellular effector of fibrotic responses and may also promote a fibrogenic phenotype in various cells, such as immune and vascular cells [42]. Toll-like receptor signaling, oxidative stress, and proinflammatory cytokines (such as IL-1β, TNF-α, and IL-6) can stimulate the transcription of TGF-β isoforms in fibrotic tissues [15, 43, 44]. Recently, Lee et al. showed that BBT-877 attenuated fibrosis in a mouse model of bleomycin-induced pulmonary fibrosis [24]. Consistently, our data also showed that TGF-β expression was significantly increased with the expression levels of fibrotic factors, including fibronectin, CTGF, and COL1A1 in diabetic mice. However, these increases in expression were significantly inhibited by BBT-877 treatment.

In conclusion, our study demonstrates that BBT-877 may reduce blood glucose levels and suppress the expression of inflammatory cytokines and fibrotic factors in the kidney, thereby improving kidney dysfunction in type 1 diabetic mice. Based on our findings, we conclude that there are two different mechanisms by which BBT-877 indirectly enhances insulin sensitivity and attenuates lipotoxicity in peripheral tissues, as well as directly suppresses inflammation and fibrosis in the kidney. Therefore, ATX inhibition could be another potential approach for LPA-LPAR antagonism and could be a potential target for therapeutic strategies to slow the progression of DN.

## MATERIALS AND METHODS

### Animals

Eight-week-old male C57BL/6N mice (*n* = 50) with similar body weights were used to establish an STZ-induced diabetic mouse model. Briefly, the mice were randomly divided into control and STZ groups, and mice in the latter group were intraperitoneally administered 50 mg/kg STZ after fasting for 4 h in the morning, once daily for 5 days. Citrate buffer (pH 4.5) was injected according to the body weight of each mouse to serve as a control. Blood glucose levels were monitored every 3 days for 2 weeks following STZ injection. Diabetes was confirmed by a blood glucose level of > 320 mg/dl. After the establishment of the STZ-induced diabetic mouse model, animals with similar body weights were randomly divided into the following groups (*n* = 8−10 mice per group): STZ-vehicle, STZ-BBT-877, and STZ-losartan. Age-matched non-diabetic mice were used as controls. BBT-877 was freshly prepared immediately before administration, according to the instructions provided by Bridge Biotherapeutics, Inc. BBT-877 was orally administered every day in the morning (09:00) and in the evening (19:00) at doses of 10 mg/kg, 30 mg/kg, and 90 mg/kg for 8 weeks. Mice in the losartan group were orally administered a dose of 10 mg/kg every day in the morning (09:00) and vehicle solution (PEG200 + 0.5% methylcellulose (400 cp) at a 1:3 volume ratio) in the evening (19:00) for 8 weeks. All procedures were approved by the Institutional Animal Care and Use Committee of the Gachon University.

### Biochemical parameters in blood and urine

Four weeks after the administration of BBT-877, losartan, or vehicle control, mice were housed in individual metabolic cages for 24 h allowing measurement of food intake, water intake, and urine volume. Urine was collected and, after debris removal via gentle centrifugation at 2,000 rpm for 5 min, stored at −80°C until analysis. All parameters, such as creatinine and microalbumin, were measured by Knotus Co., Ltd. (Incheon, Korea). Urinary neutrophil gelatinase-associated lipocalin (NGAL) was measured using a commercially available mouse ELISA kit (Abcam, ab119601) according to the manufacturer instructions. NGAL concentrations were normalized to urinary creatinine concentrations. Blood was collected via the tail vein and non-fasting glucose levels were measured using a glucose analyzer (One Touch^®^Ultra, LifeScan Johnson&Johnson, Milpitas, CA, USA) every week for 8 weeks. Hemoglobin A1c (HbA1c) was detected from blood using a DCA System HbA1c Reagent Kit (Siemens, New York, NY, USA) 8 weeks after drug treatment. Blood was also collected at the end of the experiment, immediately after sacrificing, and centrifuged at 10,000 rpm and then 5,000 rpm, each for 10 min. Finally, the supernatants were collected and stored at −80°C until analysis. The serum levels of blood urea nitrogen (BUN), creatinine, cholesterol, and triglycerides were assessed by the Southeast Medi-Chem Institute.

### Renal histological assessment

After 8 weeks of treatment with BBT-877, the mice were sacrificed and their kidneys were harvested, rapidly collected, and weighted. The left kidney was fixed in 10% formalin buffer and embedded in paraffin. Sections were stained with hematoxylin and eosin, and the glomerular structure was observed under a light microscope. The sections were also stained with the periodic acid-Schiff kit (Sigma-Aldrich, St. Louis, MO, USA), according to the manufacturer’s instructions. Images of the glomerulus were acquired under a microscope at 200× magnification. Glomerular volumes were analyzed using the ImageJ program (NIH, Bethesda, MD, USA), with up to 30 glomeruli from each mouse.

### Western blotting

Total protein was isolated from tissues using mammalian protein extract buffer (GE Life Science, 28-9712-79) containing a protease inhibitor cocktail (Sigma-Aldrich, P8340). Equal quantities of protein were separated and transferred onto polyvinylidene fluoride membranes. The membranes were then incubated with one of the following antibodies, as appropriate, at a concentration of 1 in 1,000: anti-β-actin (Cell Signaling, #8457), fibronectin (Santa Cruz, sc-8422), COL1A1 (Santa Cruz, sc-293182), CTGF (Santa Cruz, sc-101586), TGF-β (Cell Signaling, #3711), and anti-lipocalin-2/NGAL antibody (Abcam, ab63929). The density of each band was quantified using ImageJ and normalized to β-actin.

### Quantitative reverse transcriptase-polymerase chain reaction (qRT-PCR)

Total RNA was extracted from the kidney tissue of the STZ-induced diabetic mouse model using TRIzol reagent (TAKARA). cDNA was synthesized from 2 μg of total RNA using the PrimeScript 1st strand cDNA synthesis kit (TAKARA, 6110A). Real-time qRT-PCR was performed using the Applied Biosystems Prism 7900HT Real-Time PCR system. Relative gene expression levels were normalized to cyclophilin A RNA. The primers used for qRT-PCR were as follows: IL6-forward primer, 5′TCCAGTTGCCTTCTTGGGACTGAT3′, reverse primer, 5′AGCCTCCGACTTGTCAAGTGGTAT3′, MCP-1-forward primer, 5′GCAGTTAACGCCCCACTCA3′, reverse primer, 5′CCAGCCTACTCATTGGGATCA3′, TNF-α-forward primer, 5′CCAACGGCATGGATCTCAAAGACA3′, reverse primer, 5′AGATAGCAAATCGGCTGACGGTGT3′, PPAR α-forward primer, 5′TATTCGGCTGAAGCTGGTGTAC3′, reverse primer, 5′CTGGCATTTGTTCCGGTTCT3′, CPT-1-forward primer, 5′CAAAGATCAATCGGACCCTAGAC3′, reverse primer, 5′CGCCACTCACGATGTTCTTC3′, FAS-forward primer, 5′CGCCACTCACGATGTTCTTC3′, reverse primer, 5′AGAGACGTGTCACTCCTGGACTT3′, SCD-1-forward primer, 5′CCGGAGACCCTTAGATCGA3′, reverse primer, 5′TAGCCTGTAAAAGATTTCTGCAAACC3′, FABP-4 forward primer, 5′ACACCGAGATTTCCTTCAAACTG3′, reverse primer, 5′CCATCTAGGGTTATGATGCTCTTCA3′, NGAL forward primer, 5′GGCAGCTTTACGATGTACAGCA3′, reverse primer, 5′TCTGATCCAGTAGCGACAGCC3′.

### Statistical analysis

All results are expressed as mean ± SEM. Differences between more than two groups were analyzed using one-way ANOVA followed by Scheffe’s post-hoc multiple comparison test. Statistical significance was set at *p* < 0.05.

## Supplementary Materials

Supplementary Figures
