# Supplementary material for: Effect of BBT-877, a novel inhibitor of ATX, on a mouse model of type 1 diabetic nephropathy
Source: Aging (Albany NY). 2022 Aug 26;14(16):6467–80. doi: 10.18632/aging.204249 (PMC9467391; doi:10.18632/aging.204249)
Supplement: Supplementary Figures [file aging-14-204249-s001.pdf]

SUPPLEMENTARY FIGURES

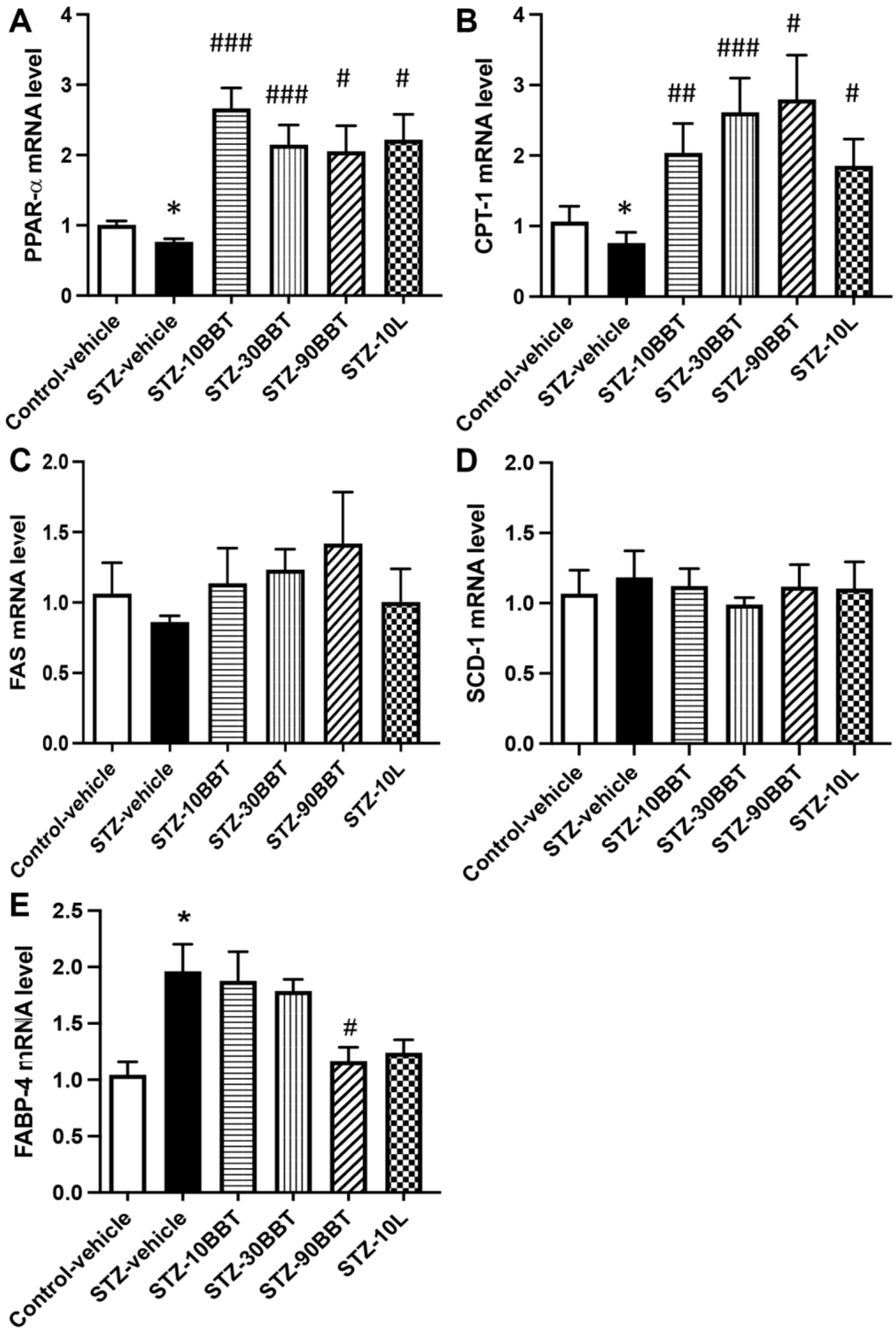

Supplementary Figure 1. BBT-877 reduces fat accumulation in the liver in STZ-induced diabetic mice. After treatment with BBT-877 for 8 weeks, mice were sacrificed, and their livers were collected. RNA was extracted and subjected to qRT-PCR for analyzing (**A**) PPAR-α, (**B**) CPT-1, (**C**) FAS, (**D**) SCD-1, and (**E**) FABP-4 expression levels. Abbreviations: STZ: streptozotocin; qRT-PCR: quantitative reverse transcriptase-polymerase chain reaction. STZ-10BBT contains 10 mg/kg of BBT-877; STZ-30BBT contains 30 mg/kg of BBT-877; STZ-90BBT contains 90 mg/kg of BBT-877; and STZ-10L contains 10 mg/kg of losartan. $^{*}p < 0.05$, control-vehicle vs. STZ-vehicle, $^{\#}p < 0.05$, STZ-vehicle vs. STZ-10, 30, 90BBT or STZ-10L, n = 8–10.

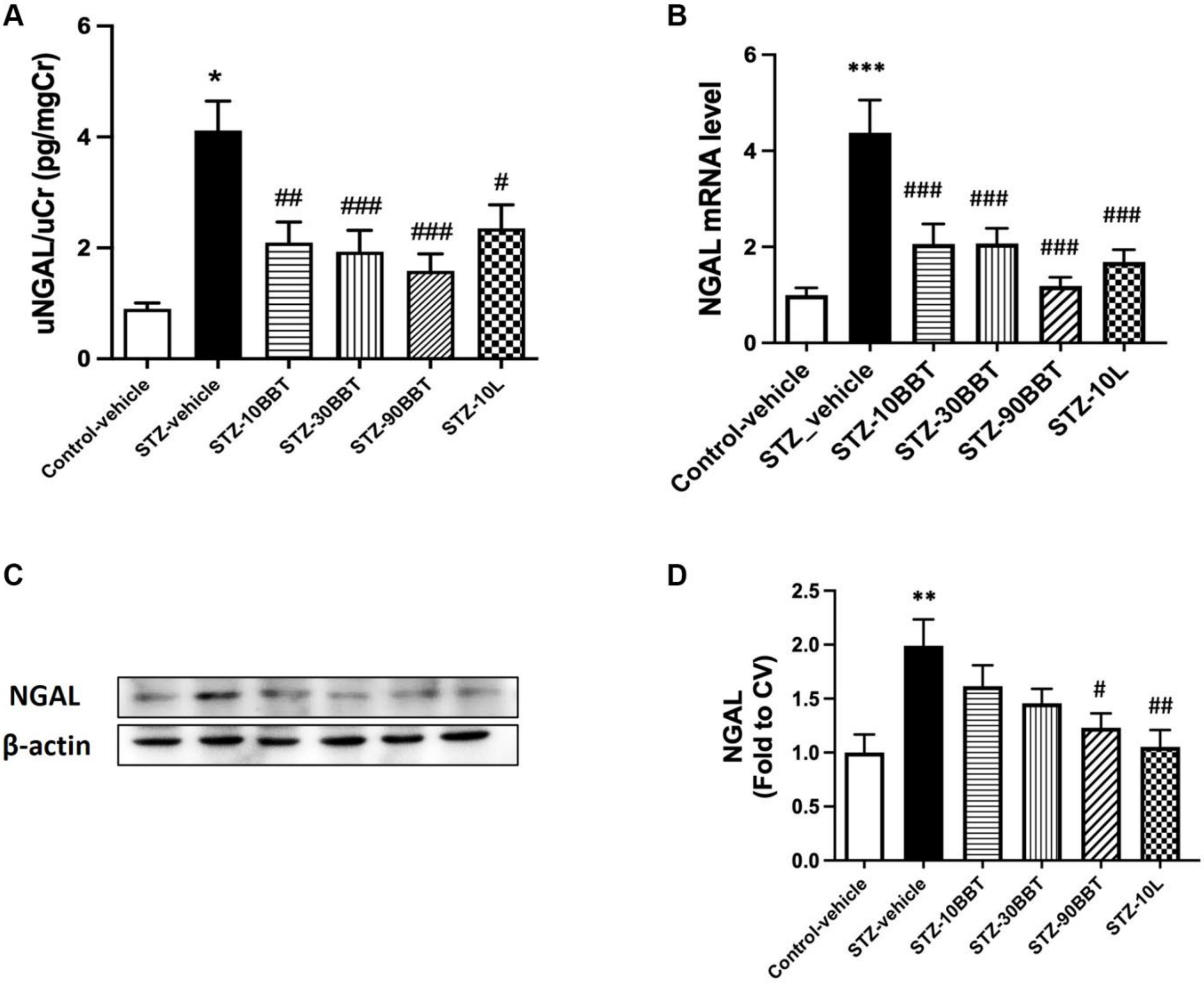

Supplementary Figure 2. BBT-877 reduces glomerular injury in the kidneys of STZ-induced diabetic mice. After treatment with BBT-877 for 8 weeks, urinary neutrophil gelatinase-associated lipocalin (NGAL) were measured. (**A**) Urinary NGAL level; (**B**) NGAL mRNA in kidney tissue; (**C**) Representative image; (**D**) Urinary NGAL protein level in kidney tissue. Abbreviation: STZ: streptozotocin. STZ-10BBT stands for BBT-877 10 mg/kg; STZ-30BBT stands for BBT-877 30 mg/kg; STZ-90BBT stands for BBT-877 90 mg/kg; and STZ-10L stands for losartan 10 mg/kg. $^{*}p < 0.05$, control-vehicle vs. STZ-vehicle, $^{\#}p < 0.05$, STZ-vehicle vs. STZ-10, 30, 90BBT or STZ-10L, n = 8−10.
